# A252 DRUG DISCONTINUATION AND PERSISTENT SYMPTOMS AMONG THE USERS OF BIOSIMILAR AND LEGACY ANTI-TNF DRUGS IN A REAL-WORLD CANADIAN IBD COHORT

**DOI:** 10.1093/jcag/gwad061.252

**Published:** 2024-02-14

**Authors:** B Oketola, L Lukusa, M Birck, A Neville, Y Leung, N Narula, W Afif, S Bernatsky, H Singh

**Affiliations:** Internal Medicine, University of Manitoba, Winnipeg, MB, Canada; McGill University, Montreal, QC, Canada; McGill University, Montreal, QC, Canada; McGill University, Montreal, QC, Canada; The University of British Columbia, Vancouver, BC, Canada; Hamilton Health Sciences, Hamilton, ON, Canada; McGill University, Montreal, QC, Canada; McGill University, Montreal, QC, Canada; Internal Medicine, University of Manitoba, Winnipeg, MB, Canada

## Abstract

**Background:**

Biosimilars and their reference originator drugs are increasingly used for inducing and maintaining remission in Inflammatory Bowel Diseases (IBD). Persistent symptoms, such as fatigue and joint pain, have been reported among individuals with IBD in remission. However, there is limited data on the persistence of symptoms among long-term users of biological therapy.

**Aims:**

We report on drug discontinuation and persistent symptoms among a multicentre cohort of IBD patients 12 months after initiating anti-TNF originators or biosimilars.

**Methods:**

We enrolled and followed individuals with IBD (ampersand:003E18 years) who initiated therapy with infliximab or adalimumab, bio-originators/biosimilars, between January 2019 and March 2023. We determined the rate of discontinuation of therapy, persistent symptoms, and quality of life (QoL) after 12 months. Difference in effect estimates (proportions and means) between the two groups (originators versus biosimilars) was determined.

**Results:**

181 individuals with IBD (CD = 58%, female = 46%, Caucasian = 85%) were enrolled. 62% were biologic-naïve at initiation. Of the 119 participants who completed a 12-month follow-up visit, about 19% (n = 34) discontinued drug use (all were infliximab users). Insufficient disease control was the most common reason for drug discontinuation (18.2%, n = 33). There was a significant reduction in the number of corticosteroid users at follow-up in both originator (rate ratio: 0.33, 95% confidence interval (CI): 0.16-0.49) and biosimilar users (rate ratio: 0.22, 95% CI: 0.14-0.30). With our limited power, we were unable to detect significant reductions in ER visits and hospitalizations in the first year after initiation of anti-TNF agents compared to that in the preceding year. The severity of pain/discomfort anywhere and the frequency of fatigue and anxiety/depression was not significantly reduced at 12 months in either of the groups. The percentage of those reporting moderate/severe abdominal pain was reduced among biosimilar users (46% to 20%; rate ratio of 0.26, 95% CI: 0.15-0.38), and originator users (28% to 13%, rate ratio 0.16, 95% CI: -0.02, 0.33). The proportion of those reporting rectal urgency at least some of the time was also reduced among both originators (30% at baseline to 9% at follow-up) and biosimilar users (33% to 17%). There was a significant improvement in QoL scores among both originator users (mean difference (MD) 13.0, 95% CI: 6.00-22.0) and biosimilar users (MD 10.0, 95% CI: 6.00-14.0) on the SIBDQ. This improvement was replicated on the EQ-5D only among biosimilar users.

**Conclusions:**

Our study results suggest that even though the use of anti-TNF drugs leads to improvement in QoL, there may be no reduction in some symptoms, such as fatigue, anxiety, depression.

**Funding Agencies:**

CIHR

